# Causes and consequences of variation in early‐life telomere length in a bird metapopulation

**DOI:** 10.1002/ece3.9144

**Published:** 2022-07-31

**Authors:** Michael Le Pepke, Thomas Kvalnes, Peter Sjolte Ranke, Yimen G. Araya‐Ajoy, Jonathan Wright, Bernt‐Erik Sæther, Henrik Jensen, Thor Harald Ringsby

**Affiliations:** ^1^ Department of Biology, Centre for Biodiversity Dynamics (CBD) Norwegian University of Science and Technology (NTNU) Trondheim Norway

**Keywords:** demography, dispersal, early‐life, individual heterogeneity, life‐history, pace‐of‐life, stress, telomere dynamics

## Abstract

Environmental conditions during early‐life development can have lasting effects shaping individual heterogeneity in fitness and fitness‐related traits. The length of telomeres, the DNA sequences protecting chromosome ends, may be affected by early‐life conditions, and telomere length (TL) has been associated with individual performance within some wild animal populations. Thus, knowledge of the mechanisms that generate variation in TL, and the relationship between TL and fitness, is important in understanding the role of telomeres in ecology and life‐history evolution. Here, we investigate how environmental conditions and morphological traits are associated with early‐life blood TL and if TL predicts natal dispersal probability or components of fitness in 2746 wild house sparrow (*Passer domesticus*) nestlings from two populations sampled across 20 years (1994–2013). We retrieved weather data and we monitored population fluctuations, individual survival, and reproductive output using field observations and genetic pedigrees. We found a negative effect of population density on TL, but only in one of the populations. There was a curvilinear association between TL and the maximum daily North Atlantic Oscillation index during incubation, suggesting that there are optimal weather conditions that result in the longest TL. Dispersers tended to have shorter telomeres than non‐dispersers. TL did not predict survival, but we found a tendency for individuals with short telomeres to have higher annual reproductive success. Our study showed how early‐life TL is shaped by effects of growth, weather conditions, and population density, supporting that environmental stressors negatively affect TL in wild populations. In addition, shorter telomeres may be associated with a faster pace‐of‐life, as individuals with higher dispersal rates and annual reproduction tended to have shorter early‐life TL.

## Graphical abstract

Our study showed how early‐life telomere length (TL) in wild house sparrows is shaped by effects of growth, weather conditions, and population density. Furthermore, TL may be associated with individual pace‐of‐life, with higher dispersal rates and annual reproduction tending to be associated with shorter early‐life TL. However, associations between environment, early‐life TL, and fitness may differ between different populations in the wild.

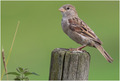

## 1 INTRODUCTION

Telomeres are short repetitive nucleotide sequences capping the ends of linear chromosomes (Blackburn & Szostak, 1984). Recent studies have shown that individual variation in telomere dynamics might play an important role shaping the life‐history of many species, including wild birds (Eastwood et al., 2019; Spurgin et al., 2018; Vedder et al., 2021), reptiles (Olsson et al., 2018a), mammals (Foley et al., 2020; van Lieshout et al., 2019), and fish (McLennan et al., 2016). Telomere shortening occurs during cell division and is accelerated by oxidative stress (Jennings et al., 1999; von Zglinicki, 2002). When telomeres become critically short, apoptosis and cellular senescence may be triggered (Aubert & Lansdorp, 2008), and TL is considered a biomarker of organismal aging (López‐Otín et al., 2013). However, individual differences in telomere length (TL) are established early in life (Entringer et al., 2018; Martens et al., 2021), and may thus reflect cumulative effects of physiological stress incurred during early life (Chatelain et al., 2020; Nettle et al., 2017; Ridout et al., 2018).

From an eco‐evolutionary perspective, individual telomere dynamics are interesting because they have been shown to be associated with survival and reproductive success in some free‐living animal populations (Chatelain et al., 2020; Fairlie et al., 2016; Froy et al., 2021; Haussmann et al., 2005; Heidinger et al., 2021; Olsson et al., 2018b; Sudyka, 2019). Furthermore, TL has been shown to predict individual health, quality, or lifespan within several species (Asghar et al., 2015; Eastwood et al., 2019; Fairlie et al., 2016; Heidinger et al., 2012; van Lieshout et al., 2019; Wilbourn et al., 2018). Covariation between TL dynamics and fitness therefore suggests that TL could act as a “causal mediator” of the life‐history trade‐offs between growth, survival, and reproduction (Heidinger et al., 2021; Monaghan, 2014; Monaghan & Haussmann, 2006; Tobler et al., 2021). Alternatively, TL may be a transient, environmentally pliant trait reflecting experienced stress or individual quality (i.e., a non‐causal biomarker,Bateson & Poirier, 2019; Boonekamp et al., 2013), but with few direct fitness consequences (Young, 2018).

Whether telomere dynamics underpin constraints in individual variation in life‐history strategies remains debated (Monaghan, 2010; Tobler et al., 2021; Vedder et al., 2017). Giraudeau, Angelier, and Sepp (2019) speculated that TL could act as a physiological mediator of the individual variation in suites of life‐history traits (pace‐of‐life syndromes, e.g., Reale et al., 2010) within species. It has also been suggested that telomere dynamics may underlie behavioral patterns or personalities (Adriaenssens et al., 2016; Bateson & Nettle, 2018; Espigares et al., 2021). However, studies have yet to identify the mechanisms underlying TL dynamics in natural populations and the potential of using TL as a biomarker of physiological costs of individual experiences, or somatic redundancy, in the wild (Bateson & Poirier, 2019; Boonekamp et al., 2013). To understand the ecological and evolutionary significance of TL it is therefore important to identify the causes and consequences of individual variation in TL.

Previous studies have shown TL to be affected by body size or growth (Monaghan & Ozanne, 2018; Pepke, Kvalnes, et al., 2022), age (Remot et al., 2021; Salomons et al., 2009), body condition (Barrett et al., 2013; Rollings, Uhrig, et al., 2017), hatch day (Beaulieu et al., 2017), and habitat quality (Angelier et al., 2013; McLennan et al., 2021; Spurgin et al., 2018; Watson et al., 2015; Wilbourn et al., 2017), or that there are sex‐differences in TL (Barrett & Richardson, 2011). Furthermore, several environmental stressors may induce oxidative stress‐mediated effects on TL, in particular harsh abiotic conditions, poor nutrition, and pathogen infection have been shown to shorten telomeres (Chatelain et al., 2020; Pepper et al., 2018). However, the link between oxidative stress and telomere shortening has rarely been demonstrated in vivo (Boonekamp et al., 2017; Reichert & Stier, 2017). Weather conditions may have direct effects on TL, for example, through thermoregulation and metabolic activity (Angelier et al., 2018), or indirect effects, for example, changes in food availability (Criscuolo et al., 2020; Spurgin et al., 2018) or pathogen prevalence (Asghar et al., 2015; Giraudeau, Heidinger, et al., 2019). Depending on the species‐specific optima and the range of weather conditions experienced there could be linear or non‐linear associations between environmental conditions and TL (Axelsson et al., 2020).

Local demography such as population density may influence the competitive regimes experienced by parents during breeding (Dhondt, 2010). In populations of house sparrows (*Passer domesticus*), density regulation affected recruit production, which generated variation in pace of life‐history strategies across populations (Araya‐Ajoy et al., 2021). However, physiological mechanisms underlying such demographic processes remain largely unknown (Edwards et al., 2021). Changes in TL dynamics may underpin physiological stress responses to changes in demography (Bergman et al., 2019; Gangoso et al., 2021). For instance, Spurgin et al. (2018) found weak evidence for a negative effect of population density on early‐life TL and telomere attrition in Seychelles warblers (*Acrocephalus sechellensis*). They also found that TL was positively associated with abundance of insects, the main food resource for the warblers, indicating that increased food availability may have masked negative effects of increased density on TL (Brown et al., 2021).

Short early‐life telomeres may be associated with a faster pace‐of‐life (Giraudeau, Angelier, & Sepp, 2019), which could involve an increased probability of exploratory behavior (Adriaenssens et al., 2016) that increases chances of dispersal (Cote et al., 2010; Dingemanse et al., 2003; Dingemanse et al., 2020). However, if dispersal is condition‐dependent (Ims & Hjermann, 2001), and the telomere–survival relationship is causal (Wilbourn et al., 2018) even in early life (Monaghan & Ozanne, 2018), short early‐life telomeres may have physiological consequences that prevent dispersal, rendering individuals with long telomeres more likely to become successful dispersers. Yet, little is known about the physiological mechanisms that could mediate suites of traits associated with dispersal (Clobert et al., 2012) and if telomere dynamics are involved (Canestrelli et al., 2020).

**FIGURE 1:**
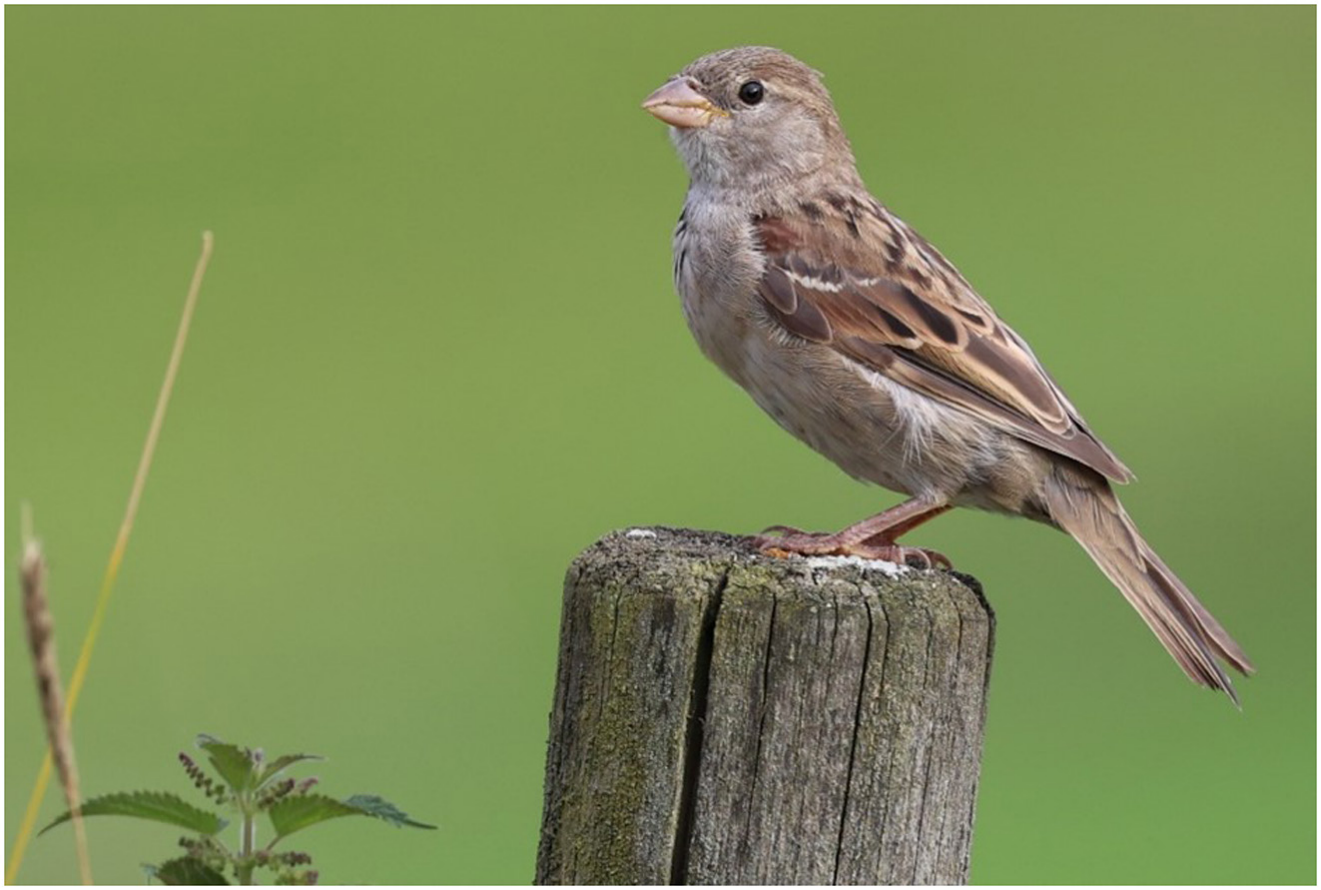
Juvenile house sparrow (*Passer domesticus*), Helgeland, Norway. Photo by P.S. Ranke.

**FIGURE 2:**
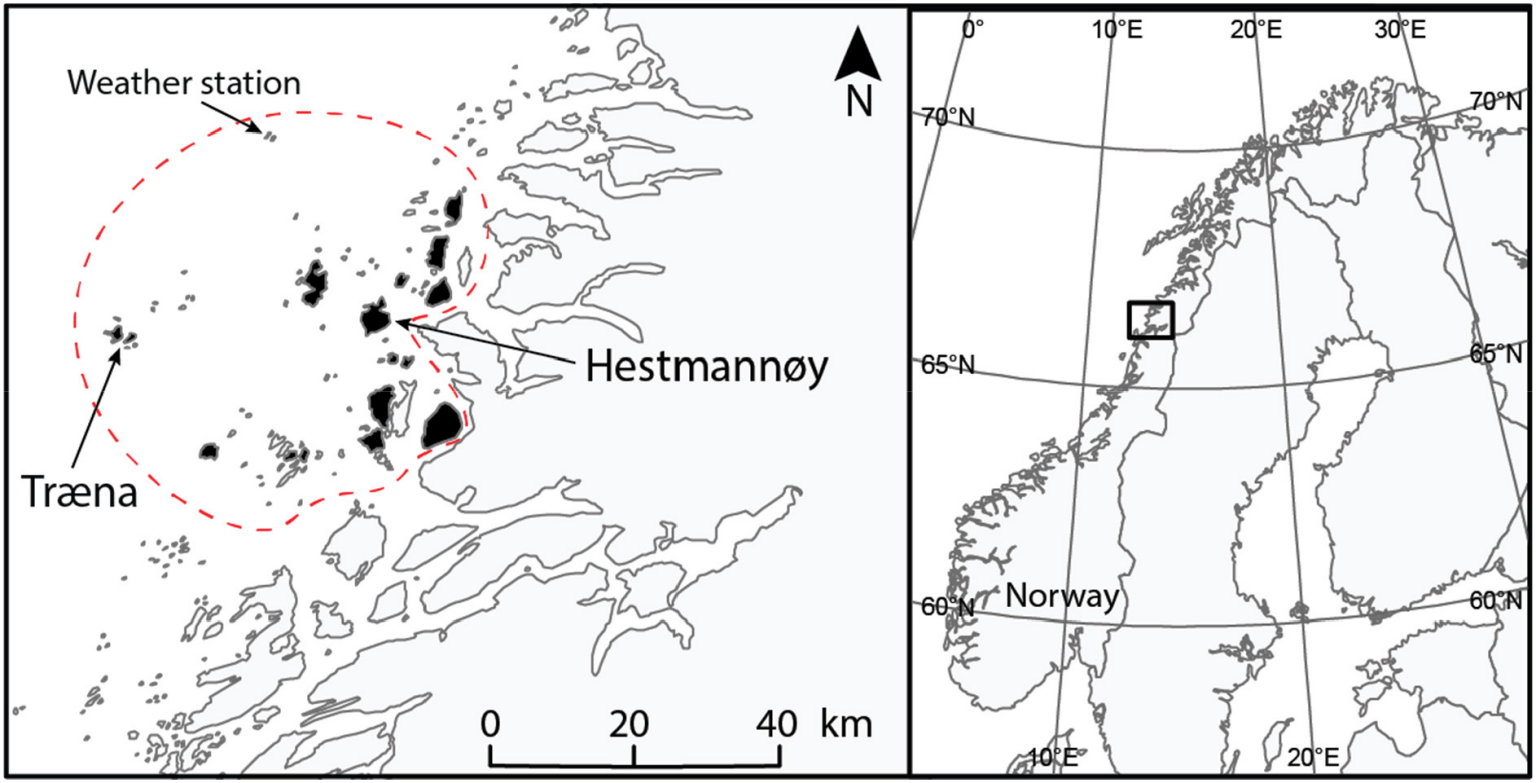
Map of the house sparrow metapopulation study area in northern Norway. We measured early‐life telomere lengths in sparrows hatched on the islands of Hestmannøy and Træna. Mist netting and observations took place regularly on the black islands, which are the main inhabited islands within the monitored study area (red dotted line). Human settlements on the mainland coast east of the study area were visited in autumn and spring to search for dispersers. Weather data was retrieved from a meteorological station at the island of Myken.

Investigating spatiotemporal variation in traits such as TL that may be involved in producing individual variation in life‐history traits therefore seems to be fundamental to a proper understanding of population ecology and life‐history evolution. In this study, we investigate causes and consequences of spatiotemporal variation in early‐life TL across two decades in two populations of wild house sparrows located within an island metapopulation study system (Figures 1 and 2). The two populations in our study occupy contrasting habitats: one farm‐living population with access to shelter and food throughout the year; and one garden‐living population that may be more exposed to weather conditions (Pärn et al., 2009). We have previously showed that there is a low heritability of early‐life TL (*h* ^*2*^ = 0.04) in this metapopulation, and that individual variation in TL is mainly driven by environmental (among year) variance, which may result in cohort effects on early‐life TL (Pepke et al., 2021). This long‐term study allows us to disentangle the effects of weather conditions during pre‐ and post‐natal stages on variation in TL. First, we investigate functional relationships between early‐life TL, fledgling body size and condition, local population density fluctuations, weather variables, and habitat type. Second, we test if early‐life TL is associated with natal dispersal within the metapopulation. Finally, we quantify consequences of variation in early‐life TL on recruitment probability, mortality risk, and reproductive success and whether these differ between habitat types.

## 2 MATERIALS AND METHODS

### 2.1 Study system and field data collection

We monitored two insular house sparrow populations; one on Hestmannøy (66°33'N, 12°50'E, 12.9 km^2^) in the years 1994–2020, and one on Træna (i.e., Husøy island, 66°30'N, 12°05′E, 1.5 km^2^) from 2004 to 2020, both located in an archipelago in northern Norway (Figure 2). These islands are characterized by heathland, mountains, and sparse forest. On Hestmannøy (“farm island”), close to the mainland, cultivated grassland (silage production and grazing) dominates the landscape, and the sparrows live closely associated with humans on dairy farms, where they have access to food (grain) and shelter (barns) all year. Most nests are found in cavities inside sheltered or heated barns. In contrast, on Træna (“non‐farm island”), ca. 34 km further out into the sea (Figure 2), there are no farms, but a small village largely consisting of detached houses. Here, the sparrows live in gardens and urban spaces, and nest in artificial nest boxes on the outside walls of the houses. Nests were visited at least every ninth day during the breeding season (May–August) to record hatch day. Fledglings were ringed with a unique color combination at around 10 days old (5–14 days) and tarsometatarsus (tarsus) was measured using calipers to nearest 0.01 mm. Body mass was measured using a Pesola spring balance to nearest 0.1 g. No morphological measurements were made for 224 nestlings (out of 2746). For 189 nestlings, blood samples and morphological measurements were not made at the same age (varying with up to ±6 days differences due to logistical reasons). We therefore fitted linear regressions of tarsus length and mass, respectively, on age (including a squared effect of age) separately for each sex and population (see Pepke et al., 2021). The measured tarsus length or mass was then adjusted to the predicted length at the age of blood (TL) sampling using the fitted values from the regressions. Nestling body condition was then calculated as the residuals of a linear regression of log_10_‐transformed mass against log_10_‐transformed tarsus length (i.e., body mass corrected for structural body size, Schulte‐Hostedde et al., 2005). Because tarsus length increases with nestling age, we used the residuals from a regression of tarsus length on age and age squared as a measure of age‐corrected (age‐standardized) tarsus length (see Pepke, Kvalnes, et al., 2022). Birds were observed or captured using mist nets during summer and autumn (May–October). Blood samples (25 μl) were collected by venipuncture and stored in 96% ethanol at room temperature in the field and at −20°C in the laboratory until DNA extraction.

### 2.2 Molecular methods

Molecular sexing and microsatellite pedigree construction for this study was carried out as described in Jensen et al. (2003) and Rønning et al. (2016). Genetic pedigrees were reconstructed for individuals born or captured from 1993 to 2013. The sampling of nestlings included 1314 males, 1348 females, and 84 individuals of unknown sex (total *n* = 2746). Relative TLs of DNA extracted from whole blood (mainly erythrocytes) were measured on 70–90% of the nestlings (5–14 days old) ringed each season on Hestmannøy in the years 1994–2013 (*n* = 2110, 20 cohorts) and Træna from 2004 to 2013 (*n* = 636, 10 cohorts, Table S1). DNA extraction is described in Pepke, Kvalnes, et al. (2022). All samples had DNA concentrations >15 ng μl^−1^ and an acceptable 260/280 absorbance ratio of 1.8–2.2. DNA was diluted with distilled H_2_O to 1.67 ng μl^−1^ and stored at −78°C. Relative TLs (T/S ratios) were measured using the qPCR method (following Cawthon, 2002; Criscuolo et al., 2009) as described in Pepke, Kvalnes, et al. (2022), Pepke, Niskanen, et al. (2022) and validated by Ringsby et al. (2015). Telomeric DNA was amplified using real‐time qPCR and the telomere repeat copy number was estimated relative to an invariant control gene (GAPDH, Atema et al., 2013) and a reference sample. All reactions were carried out by the same person (MLP) to avoid inter‐individual measurement variability. All samples were randomized across 125 96‐well plates. Assays were prepared using the Absolute blue qPCR SYBR green Low Rox master mix (ThermoFisher Scientific) and run on a Stratagene Mx3005p system. Telomere primers Tel1b (5′‐CGG TTT GTT TGG GTT TGG GTT TGG GTT TGG GTT TGG GTT‐3′) and Tel2b (5′‐GGC TTG CCT TAC CCT TAC CCT TAC CCT TAC CCT TAC CCT‐3′) were prepared at a final concentration of 500 nM. GAPDH primers (forward primer 5′‐GAG GTG CTG CTC AGA ACA TTA T‐3′ and reverse primer 5’‐ACG GAA AGC CAT TCC AGT AAG‐3′) were prepared at a final concentration of 200 nM. Telomere assay thermal profile was: 15 min at 95°C, 27 cycles of 15 s at 95°C, 30 s at 58°C, and 30 s at 72°C. GAPDH thermal profile was: 15 min at 95°C, 40 cycles of 15 s at 95°C, 15 s at 60°C. Assays were followed by melt curve analysis (58–95°C 1°C/5 s ramp) and checked for a single peak dissociation curve. Mean telomere assay efficiency was 97.5 ± 3.9%, and mean GAPDH assay efficiency was 97.6 ± 4.2%. Average reference sample cycle thresholds across all plates were 10.54 ± 0.03 SD for telomere assays and 21.53 ± 0.02 SD for GAPDH assays. Thus, TL measurement reproducibility within the same (reference) sample was high across plates, and re‐extractions of the same blood samples followed by runs on different plates revealed highly correlated TL measurements (*R* ^2^ = 0.75, see details in Pepke et al., 2021). Data were analyzed using the qBASE software (Hellemans et al., 2007) controlling for inter‐run variation.

### 2.3 Factors affecting early‐life telomere length

To examine factors that influence individual variation in TL in house sparrow nestlings (response variable, *n* = 2456 excluding individuals with missing morphological measurements [*n* = 224] and/or missing sex [*n* = 84]), we constructed 27 candidate linear mixed effects models (LMMs) with a Gaussian error distribution fitted with maximum likelihood (ML) using the package *lme4* (Bates et al., 2015) in R v. 3.6.3 (R Core Team, 2020). The models were compared using Akaike's information criterion (Akaike, 1973) corrected for small sample sizes (AICc, Hurvich & Tsai, 1989) to identify the models best underpinned by the data. Sex and island identity (Hestmannøy or Træna) were included as fixed effects in all models, including combinations of age (number of days since hatching), age‐corrected tarsus length, body condition (defined above), hatch day (mean centered ordinal day of the year), population density (spring pre‐breeding census in the hatch year mean centered within populations), and an interaction term between population density and island identity. TL was log_10_‐transformed for normalization of residuals. To account for the possible non‐independence and temporal heterogeneity in broods and cohorts, random intercepts for brood identity (*n* = 947, nested under hatch year) and hatch year (cohort identity, *n* = 20) were included in all models. Models were validated visually using diagnostic plots and all model parameters are from models refitted with restricted maximum likelihood (REML).

### 2.4 Effects of weather on early‐life telomere length

We compiled data on daily mean temperature (K), total daily amount of precipitation (mm) and mean daily atmospheric pressure (hPa) from the nearest weather station at the island of Myken (Figure 2, around 30 km from both populations) from The Norwegian Meteorological Institute (2018). The daily North Atlantic Oscillation (NAO) index was retrieved from the National Oceanic and Atmospheric Administration (2019). The effects of weather conditions on TL were analyzed using a sliding window approach (van de Pol et al., 2016) to determine the best weather predictors within a range of time frames leading up to the TL measurement. TL was measured in nestlings at around 10 days after hatching, which had been preceded by a continuous incubation time of up to 14 days that often begins after laying of the penultimate egg (Anderson, 2006). The approximate time from conception to TL measurement is therefore around 30 days, which was used as the maximum relative timeframe (days before individual TL measurement date) for relevant weather factors affecting TL. We used the R package *climwin* and its dependencies (Bailey & van de Pol, 2016) to identify the optimal time frame during which TL is most sensitive to weather effects. This approach also allowed identifying the best descriptive weather metric (mean, maximum, minimum, or sum across the time frame to reflect cumulative environmental effects on TL) and type of relationship (linear or quadratic) between TL and the weather variable (temperature, precipitation, pressure, and the NAO index). This was specified in the model as: cinterval = “day”, range = c(30, 0), type = “relative”, stat = c(“mean”,”max”,”min”,”sum”). Analyses using minimum daily precipitation were not included since this variable would too often be zero within multiday timeframes, which prevented model convergence. All possible timeframes for each weather metric and relationship were then compared using AICc (van de Pol et al., 2016). As the baseline model (without climate effects) we used the best model of non‐weather factors affecting early‐life TL (*n* = 2462) identified from the analyses described above. Weather variables are correlated across the study system (Ringsby et al., 2002), but the microclimate may differ between the two structurally different habitats (Hestmannøy and Træna). We therefore also tested models including an interaction term between island identity and the respective weather variable. In total, 60 models were compared using AICc (Table S2). Hatch year and nested brood identity were included as random intercepts in all models. We tested for over‐fitting by randomizing data and re‐running the analyses 100 times using the *randwin* and *pvalue* functions provided in *climwin* (Bailey & van de Pol, 2016).

A positive summer NAO is often associated with warmer and drier weather in northwestern Europe (Bladé et al., 2012; Folland et al., 2009). To understand the relationship between the NAO index and local weather conditions (Stenseth et al., 2003), we tested for intercorrelation among all four weather variables (Table S2) within the total time frame actually included in the analyses (effectively between April 4, corresponding to 30 days before the earliest nestling sampling date until the last sampling date of August 19, from 1994 to 2013). This showed that a high daily NAO index primarily reflects a high daily amount of precipitation (Pearson's *r* = 0.13, *p* < .0001) during spring and summer in this area of the Norwegian coast. However, high daily amounts of precipitation were also negatively correlated with mean daily temperature and atmospheric pressure (Table S2).

### 2.5 Does early‐life telomere length predict natal dispersal?

House sparrows generally show strong site fidelity and dispersal occurs mainly among juveniles in the autumn (i.e., natal dispersal, Altwegg et al., 2000) and over short distances (Anderson, 2006; Tufto et al., 2005). All islands surrounding Hestmannøy and Træna and the inhabited areas on the mainland shores (Figure 2) were visited regularly to identify dispersers (Ranke et al., 2021; Saatoglu et al., 2021). To reduce effects of any selective disappearance of certain phenotypes before registration of dispersal, only individuals that survived until the following spring (i.e., recruits), were included in the analyses. A total of 41 individuals (18 [6 males, 12 females] out of 342 from Hestmannøy and 23 [14 males, 9 females] out of 113 from Træna) were observed on islands different from their natal islands within their first year of life (out of *n* = 455 recruits). We used logistic regression with a binomial error distribution (using the “bobyqa” optimizer throughout to facilitate model convergence, Bates et al., 2014) to test if early‐life TL predicts the probability of successful natal dispersal. Within this house sparrow metapopulation, dispersal is female‐biased and dispersal rates depend on habitat type (Ranke et al., 2021; Saatoglu et al., 2021). We therefore included sex and island identity as covariates in explaining dispersal propensity in all models. Hatch year was included as random intercept. We also included two‐ and three‐way interactions between TL, sex, and island identity to test for differing relationships between TL and dispersal across sexes and island types. With this approach, a total of nine candidate models were compared using AICc.

### 2.6 Fitness consequences of variation in early‐life telomere length

We used three approaches to investigate the consequences of variation in early‐life TL on fitness (survival and reproduction). First, we tested if TL predicts whether an individual survives its first year (*n* = 445, excluding individuals with missing tarsus length measurements) or not (*n* = 2017), that is, recruitment probability, using a logistic regression with a binomial error distribution and a logit link function (*lme4* package). Explanatory variables were TL, tarsus length, non‐linear effects of TL (TL^2^) and tarsus length (tarsus length^2^), and interaction terms between island identity and the linear effects of tarsus length and TL, respectively. Sex and island identity were included as fixed effects, and year and nested brood identity as random intercepts, in all models. A total of 14 candidate models were constructed.

Second, we used Cox proportional hazards regression to test whether TL predicted mortality risk over the lifespan using the *survival* package (Therneau, 2015). The last observation of an individual was used as an estimate of minimum lifespan (number of days since hatching). Birds were assumed to have died if they had not been observed during two subsequent field seasons. Only two individuals (out of *n* = 2462) may still have been alive when observations ended (autumn 2020) and were therefore right‐censored (Cox, 1972). We constructed the same 14 candidate models as in the first‐year survival analyses above. Brood identity was included as a random effect (cluster) and model assumptions were tested using the Schoenfeld test. To meet model assumptions, data was stratified by island identity, allowing for different hazard functions within each population (strata). The *simPH* package was used to simulate and plot the effects of the predictor variables on the hazard ratios (Gandrud, 2015). Finally, we used the Kaplan–Meier method to construct cumulative survival curves (*survminer* package, Kassambara et al., 2020).

Third, we tested if TL predicts annual reproductive success (ARS; the number of recruits [fledglings that survived until the following spring] produced per year by an individual) among individuals that survived their first year and were thus able to breed (starting from year 1995). Genetic parenthood data was not available after 2013, so subsequent years were excluded from the analysis. We fitted generalized LMMs with a Poisson distribution using the package *glmmTMB* (Brooks et al., 2017) to test whether TL predicts ARS (*n* = 709 annual reproductive events of *n* = 396 individuals). Tarsus length and non‐linear effects of TL and tarsus length were included in 14 candidate models (same as described above). All models included sex and island identity as fixed factors, and individual identity (*n* = 396) and year (*n* = 19) as random intercepts. Models were validated using the DHARMa package (Hartig, 2020). The 14 candidate models within each of the three approaches above were compared using AICc.

## 3 RESULTS

### 3.1 Factors affecting early‐life telomere length

**FIGURE 3:**
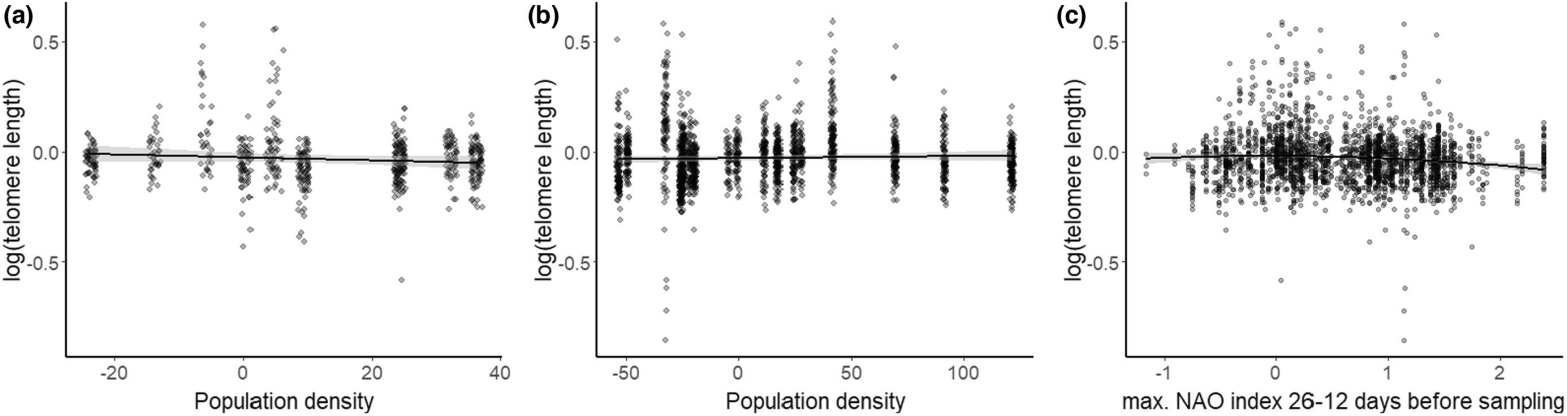
The effect of population density (mean centered) on log_10_‐transformed early‐life telomere length (TL) in (a) the Træna population (negative association) and (b) in the Hestmannøy population (no association), see Tables 1 and 2. (c) The negative quadratic association between early‐life TL and the best weather variable predictor (max. NAO index during incubation) from a sliding window analysis (Tables S2 and S3).

There was considerable variation in TL among cohorts with no obvious directional trend (Figure S1). The best model of variation in TL included a negative effect of tarsus length (*β*_tarsus_ = −0.0038 ± 0.0016, CI = [−0.0079, −0.0006], Tables 1 and 2) indicating that larger individuals had shorter telomeres. The model also included evidence for an interaction term between population density and island identity (*β*_island*density_ = 0.0008 ± 0.0004, CI = [0.4E‐4, 0.0016], *β*_density_ = −0.0008 ± 0.0004, CI = [−0.0015, −0.5E‐4]), indicating that individuals born in years with higher population densities had shorter telomeres, but only in the Træna (non‐farm) population (Figure 3a). Thus, there was apparently no evidence for an effect of variation in population density on TL in the Hestmannøy population (Figure 3b). The second‐best model (*∆*AICc = 0.4, Table 1) did not include the effects of population density. Some of the models within 1.0 < *∆*AICc <2.0 included hatch day, condition or age (Table 1), but these effects were close to 0 and had large CIs overlapping 0.

**TABLE 1:** Linear mixed effects models with ∆AICc ≤ 4 of variation in early‐life telomere length (TL) in house sparrow nestlings from two island populations.

Model		∆AICc	df	w
1	TL = sex + island + tarsus + density + island*density	0.0	9	0.1405
2	TL = sex + island + tarsus	0.4	7	0.1138
3	TL = sex + island + tarsus + density + island*density + hatch day	1.0	10	0.0872
4	TL = sex + island + tarsus + density + island*density + condition	1.1	10	0.0814
5	TL = sex + island + tarsus + density + island*density + age	1.1	10	0.0793
6	TL = sex + island + tarsus + condition	1.5	8	0.0652
7	TL = sex + island + tarsus + age	1.6	8	0.0619
8	TL = sex + island + tarsus + density + island*density + age + hatch day	1.9	11	0.0535
9	TL = sex + island + tarsus + density	2.2	8	0.0460
10	TL = sex + island + tarsus + density + island*density + condition + age	2.2	11	0.0457
11	TL = sex + island + tarsus + density + island*density + condition + age + hatch day	3.0	12	0.0311
12	TL = sex + island + density + island*density	3.2	9	0.0284
13	TL = sex + island + tarsus + density + condition	3.4	9	0.0261
14	TL = sex + island + tarsus + density + age	3.5	9	0.0249
15	TL = sex + island + hatch day	3.5	7	0.0247
16	TL = sex + island	4.0	6	0.0191

*Note*: All models included random intercepts for year and brood identity. Models are ranked by AICc, and number of degrees of freedom (df) and model weights (w) are shown.

**TABLE 2:** Estimates (*β*) with standard errors (SE) and lower and upper 95% confidence intervals (CI) from a linear mixed effects model of variation in telomere length (TL, *n* = 2456).

Response variable: TL	*β*	SE	Lower CI	Upper CI
Intercept	−0.0205	0.0133	−0.0466	0.0053
Sex (female)	−0.0041	0.0041	−0.0121	0.0039
Island (Hestmannøy)	−0.0086	0.0093	−0.0269	0.0094
*Tarsus*	*−0.0038*	*0.0016*	*−0.0070*	*−0.0006*
*Density*	*−0.0008*	*0.0004*	*−0.0015*	*−0.5E−4*
Island (Hestmannøy)*density	*0.0008*	*0.0004*	*0.4E−4*	*0.0016*
*σ* ^2^_brood ID_ (*n* = 947)	0.0036		0.0029	0.0043
*σ* ^2^_year_ (*n* = 20)	0.0020		0.0010	0.0039
Marginal *R* ^2^/conditional *R* ^2^: 0.007/0.410				

*Note*: The model included random intercepts for brood identity and year. Italics indicate parameters with CIs not overlapping zero.

### 3.2 Effects of weather on early‐life telomere length

The best model (ΔAICc = −13.49 compared to a model without weather effects, Table S2 and Figure S2) identified from the sliding window analysis of weather variables included a negative quadratic effect of the maximum NAO index during 26 to 12 days before TL sampling (Table 3 and Figure 3c), which corresponds approximately to the timing of the incubation phase. This suggests that there is a set of (optimal) environmental conditions, reflected by intermediate values of the maximum NAO index during incubation, that result in the longest telomeres in fledglings. The model output was unlikely to be a result of overfitting (*p* = .001, see Figure S2). The second‐best model, which differed by ΔAICc = −12.82, included only a linear negative effect of maximum NAO during approximately the same time window (30 to 16 days before sampling, Table S2).

**TABLE 3:** Best model identified from sliding window analyses (Table S2) of the effect of weather variables on telomere length (TL) in house sparrow fledglings (*n* = 2462).

Response variable: TL	*β*	SE	Lower CI	Upper CI
Intercept	−0.0049	0.0138	−0.0321	0.0220
Sex (female)	−0.0052	0.0041	−0.0131	0.0028
Island (Hestmannøy)	−0.0125	0.0092	−0.0305	0.0054
*Tarsus*	−*0.0042*	*0.0016*	−*0.0074*	−*0.0011*
*Density*	−*0.0009*	*0.0004*	−*0.0016*	−*0.0002*
Island (Hestmannøy)*density	*0.0009*	*0.0004*	*0.0002*	*0.0016*
max. NAO_26–12 days_	0.0124	0.0084	−0.0040	0.0287
(*max. NAO*_*26–12 days*_)^2^	*−0.0223*	*0.0052*	*−0.0325*	*−0.0121*
*σ* ^2^_brood ID_ (*n* = 948)	0.0033		0.0026	0.0040
*σ* ^2^_year_ (*n* = 20)	0.0022		0.0011	0.0042
Marginal *R* ^2^/conditional *R* ^2^: 0.029/0.418				

*Note*: Italics indicate parameters with CIs not overlapping zero.

### 3.3 Does early‐life telomere length predict natal dispersal?

**FIGURE 4:**
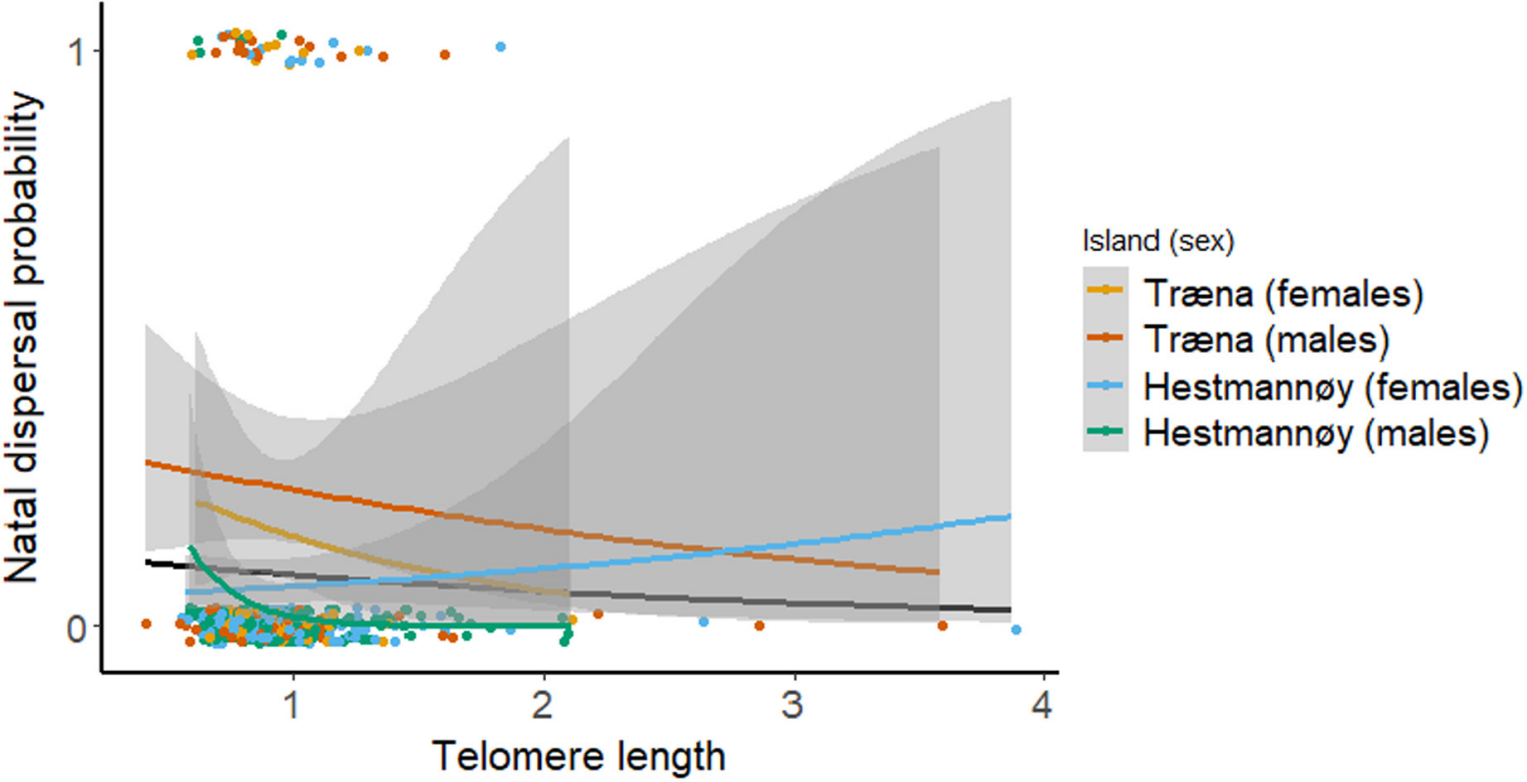
Binomial logistic regression of successful natal dispersal probability predicted by early‐life telomere length (TL, *n* = 455). The highest ranked models (Table S4) suggested a weak negative association between dispersal probability and TL (black regression line). One of these top models suggested that there was a stronger negative association between TL and dispersal probability among males born on Hestmannøy (*n* = 167, green regression line with 95% confidence intervals in gray areas).

Four of the six models with ΔAICc < 2 describing variation in successful natal dispersal probability included a tendency for a negative association between TL and dispersal probability (model ranked second with ΔAICc = 0.0; *β*_TL_ = −0.795 ± 0.630, CI = [−2.248, 0.268], Table S4 and Figure 4). The two highest ranked models (both ΔAICc = 0.0) included an interaction between island and sex, indicating a tendency for males from Træna to be more likely to disperse than males from Hestmannøy (*β*_island (Hestmannøy)*sex (female)_ = 1.196 ± 0.713, CI = [−0.189, 2.659], *β*_island (Hestmannøy)_ = −2.434 ± 0.558, CI = [−3.526, −1.341], *β*_sex (female)_ = −0.496 ± 0.497, CI [−1.512, 0.472]). The model ranked third (ΔAICc = 0.6) included a three‐way interaction term between TL, island identity, and sex, suggesting that the negative association (tendency) between dispersal probability and TL was strongest in males from Hestmannøy (*β*_TL*island (Hestmannøy)*sex (male)_ = −3.049 ± 1.765, CI = [−6.787, 0.189], see full model in Table S5 and the effect in Figure 4).

### 3.4 Fitness consequences of early‐life telomere length

**FIGURE 5:**
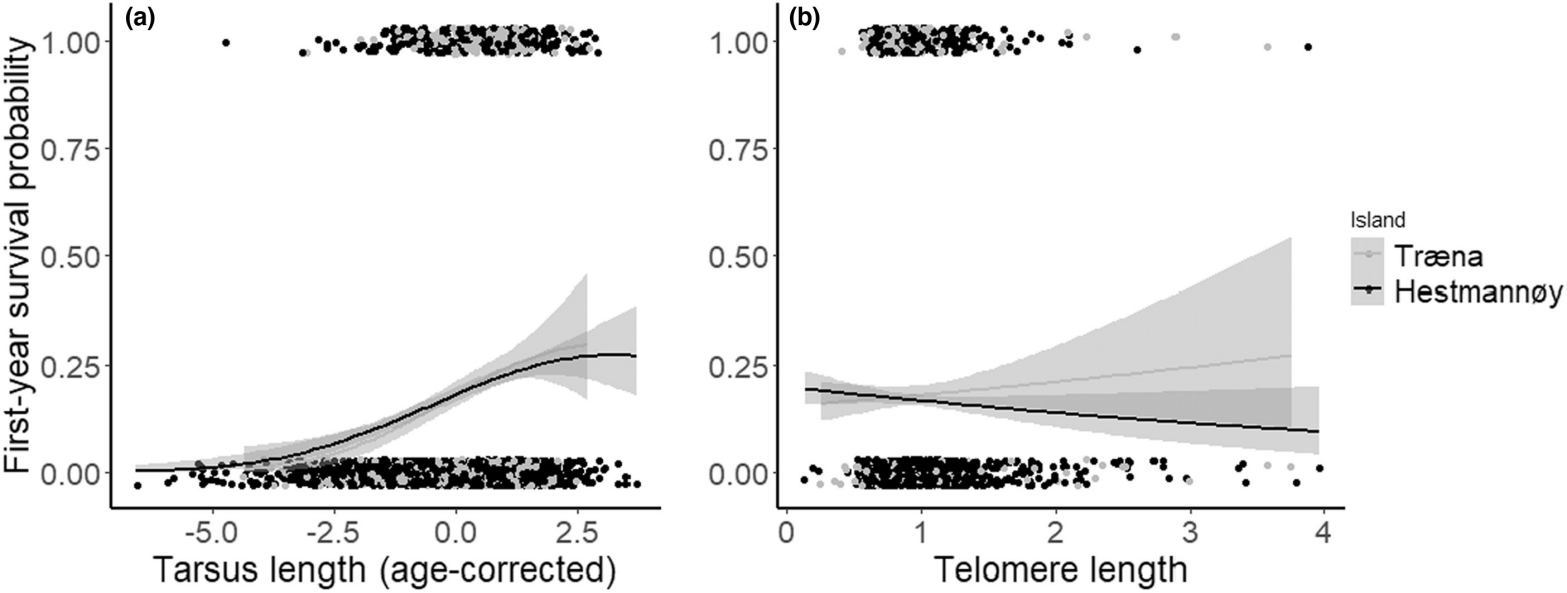
Relationship between first‐year survival (recruitment) probability in two populations of house sparrows (*n* = 2462, gray: Træna, black: Hestmannøy) and (a) fledgling tarsus length (negative quadratic association) and (b) fledgling telomere length (no evidence for any associations). The logistic regression lines are from the top models shown in Table S6 including tarsus length (model ranked 1) and telomere length (model ranked 4). There was no evidence for differences in first‐year survival probability between the two populations.

There was no evidence of an effect of TL on first‐year survival (Table S6 and Figure 5b). There was however evidence for a positive association between tarsus length and first‐year survival probability in all top models with ΔAICc <2 (model ranked 1: *β*_tarsus_ = 0.040 ± 0.009, CI = [0.057, 0.023], Table S6). The best model also included a weak curvilinear effect of tarsus length (*β*_tarsus^2_ = −0.042 ± 0.029, CI = [−0.101, 0.11]), indicating that survival probability increased less or even decreased with tarsus length in the largest individuals (Figure 5a).

**FIGURE 6:**
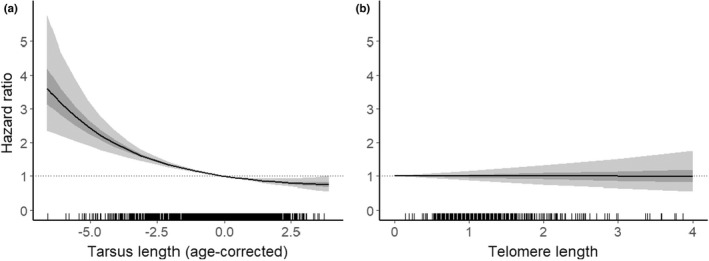
Mortality risk measured as hazard ratio in two populations of house sparrows (*n* = 2462, gray: Træna, black: Hestmannøy) as a function of (a) fledgling tarsus length (positive quadratic association) and (b) fledgling telomere length (no evidence for any associations). The simulated regression lines (black) show the modeled effect from the top models in Table S7 with 95% and 50% confidence intervals in light gray and dark gray, respectively.

There was no evidence of an effect of TL on mortality risk (Table S7 and Figure 6b). The Cox hazard regression analyses showed however that there was a strong negative association between tarsus length and mortality risk (model ranked 1: *β*_tarsus_ = −0.120 ± 0.017, CI = [−0.157, −0.083], Table S7). The best model also included a weak curvilinear effect of tarsus length (*β*_tarsus^2_ = 0.011 ± 0.006, CI = [−0.002, 0.024]), indicating that the decrease in the risk of mortality with increased tarsus length reached a plateau at large values (Figure 6a).

**FIGURE 7:**
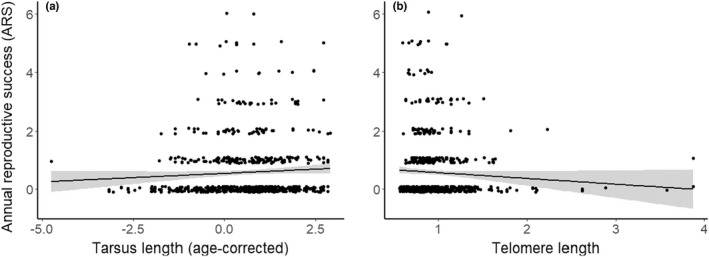
The associations between annual recruit production (ARS: annual reproductive success, *n* = 709 annual reproductive events of *n* = 396 individuals) and (a) fledgling tarsus length and (b) fledgling telomere length. The regressions lines (black, with 95% confidence intervals in gray) show the non‐significant tendencies (see the main text) predicted from the top models in Table S8.

We found weak evidence of an inverse relationship between TL and ARS (model ranked 1: *β*_TL_ = −0.446 ± 0.275, CI = [−0.985, 0.092], *n* = 709, Table S8 and Figure 7b), indicating that individuals with long TL had lower ARS than individuals with short TL. The second ranked model (∆AICc = 0.1) additionally included a weak positive effect of tarsus length on ARS (*β*_tarsus_ = 0.106 ± 0.075, CI = [−0.042, 0.253], Figure 7a). It was thus difficult to separate models including a positive effect of tarsus length and/or a negative effect of TL on ARS (Table S8).

## 4 DISCUSSION

In this study, we have shown how individual variation in early‐life TL is related to structural growth, weather conditions during incubation, and population density in a long‐term study of two island populations of wild house sparrows. This suggests a mechanistic link between environmental change and telomere dynamics in early‐life (Chatelain et al., 2020; Giraudeau, Angelier, & Sepp, 2019). TL has been shown to be associated with important components of fitness in some wild species (Eastwood et al., 2019; Froy et al., 2021; van Lieshout et al., 2019; Wilbourn et al., 2018; Young et al., 2021). However, we found little evidence that variation in TL in early life had any fitness consequences in terms of survival, but there was a tendency for a negative effect of TL on reproductive success (Tables S6–S8). Instead, fitness was mainly determined by body size (Ringsby et al., 1998) with larger individuals having higher short‐term survival (Figure 5a), lower long‐term mortality (Figure 6a), and somewhat higher reproductive success (Figure 7a). Larger individuals had shorter telomeres (Table 2), as documented previously in several species (Monaghan & Ozanne, 2018), including house sparrows (Pepke et al., 2021; Pepke, Kvalnes, et al., 2022; Ringsby et al., 2015), but little of the residual variation in fitness appeared to be explained by TL.

Recent studies have established early‐life environmental conditions as important drivers of TL dynamics in free‐living organisms (Angelier et al., 2018; Chatelain et al., 2020; Foley et al., 2020; Herborn et al., 2014; Nettle et al., 2015; Spurgin et al., 2018). Thermoregulatory and nutritional stress may increase oxidative stress, resulting in telomere shortening (Friesen et al., 2021; Reichert & Stier, 2017). Effects of weather conditions on telomere dynamics is known from other wild animal populations, including dark‐eyed juncos (*Junco hyemalis*), in which females experienced greater telomere loss during breeding at colder temperatures, probably due to cold stress (Graham et al., 2019). In black‐tailed gulls (*Larus crassirostris*), telomeres were even elongated during an El Niño year, in which weather was generally milder and sea surface temperatures lower, resulting in improved foraging conditions (Mizutani et al., 2013). Similarly, the change in TL in greater‐eared bats (*Myotis*, Foley et al., 2020), early‐life TL in European badgers (*Meles*, van Lieshout et al., 2021) and purple‐crowned fairy‐wrens (*Malurus coronatus*, Eastwood et al., 2022) was positively associated with generally good weather conditions (favorable temperatures and rainfall). Furthermore, in other Norwegian populations of house sparrows, nestling TL was positively associated with the NAO index averaged across approximately 2 weeks before and after hatching, which locally reflected higher temperatures, lower rainfall, and lower wind speed (Pepke, Kvalnes, et al., 2022). In some species of fish and lizards, higher temperatures led to shorter telomeres, which could be caused by heat stress, but also increased growth (Debes et al., 2016; Dupoué et al., 2017; Simide et al., 2016). However, in other fish and lizard species, TL increased with higher temperature (Fitzpatrick et al., 2019; Rollings et al., 2014) or showed no effect of temperature (Kim et al., 2019; McLennan et al., 2018). Furthermore, Axelsson et al. (2020) documented a thermal optimum associated with long telomers in sand lizards (*Lacerta agilis*). These idiosyncratic patterns demonstrate how environmental factors and degree of harshness may trigger a physiological stress response (Chatelain et al., 2020) with different consequences on TL dynamics depending on the deviation from species‐specific environmental optima (Axelsson et al., 2020; Friesen et al., 2021; McLennan et al., 2016; Olsson et al., 2018a).

In our study, we observed a similar curvilinear association between fledgling TL and the maximum NAO index during the incubation phase, suggesting that this weather variable best reflects the effects of environmental conditions on TL, and that there are optimal environmental conditions that result in the longest TL. A 2‐week period corresponds to the summer NAO life cycle (see Feldstein, 2007), and the maximum summer NAO may reflect extreme weather events such as drought or flooding (Drouard et al., 2019; Folland et al., 2009). At our study site, the daily NAO index was primarily positively correlated with the daily amount of precipitation (Table S2). Rainfall may affect food availability and nest attendance (Bambini et al., 2019) and hence incubation temperature (Simmonds et al., 2017), which can elicit a stress response in the organism with effects on TL (Dupoué et al., 2020; Stier et al., 2020; Vedder et al., 2018). Prenatal exposure to environmental stressors can also have significant negative effects on embryonic TL (Entringer et al., 2011; Noguera & Velando, 2019). Variation in the NAO index locally captures complex associations between weather variables reflecting “harsh” or “benign” weather conditions (Folland et al., 2009; Stenseth et al., 2003), and it has been linked to morphological and demographic changes in several northern hemisphere species (Hallett et al., 2004; Ottersen et al., 2001; Stenseth et al., 2002; Stenseth et al., 2003). For instance, the NAO index may reflect insect abundance and phenology (Nott et al., 2002; Welti et al., 2020; Westgarth‐Smith et al., 2012). The NAO can have considerable lagged effects on weather (Halkka et al., 2006), or there may be developmental time lags between weather conditions and the response in insect abundance (Visser et al., 2006). Thus, the effect of NAO during incubation may be acting on food availability during the important nestling growth stage. Food availability was positively associated with TL and TL lengthening in Seychelles warblers (Brown et al., 2021; Spurgin et al., 2018), but negatively associated with TL in American black bears (*Ursus americanus*, Kirby et al., 2017). In African striped mice (*Rhabdomys pumilio*), TL decreased during the dry season, when food availability was low, and increased during the wet season, when food availability was high (Criscuolo et al., 2020). Such associations may be complicated by the fact that some level of food restriction may reduce oxidative damage during growth (Noguera et al., 2011). Accordingly, the curvilinear effect of weather conditions on TL (Table 3) may therefore also reflect the growth conditions optimizing TL (Monaghan & Ozanne, 2018), but longitudinal TL data will be required to investigate this. Furthermore, longitudinal TL studies may help researchers to better identify the relevant timeframe for when weather affects TL most during early life.

Habitat quality may be an important driver of differences in TL dynamics across populations (Kärkkäinen, Laaksonen, et al., 2021; McLennan et al., 2021; Wilbourn et al., 2018). We found evidence for an interaction effect between habitat type (island) and population density (Table 2), suggesting that pre‐breeding population density was negatively related to TL on the non‐farm island (Træna), but not on the farm‐island (Hestmannøy). On Hestmannøy, which holds a larger sparrow population than Træna (Figure S1 and Table S1), the sparrows live and nest in a sheltered environment around farms, in contrast to Træna, where they nest in nest boxes in a village environment. House sparrows are gregarious but exhibit territorial behavior by defending nest sites during the breeding season (Anderson, 2006). Thus, there may be more competition for nest sites on Træna compared to Hestmannøy at high population densities. Furthermore, as population density increases, competition increases, and poorer quality nest and foraging sites are increasingly occupied (Møller et al., 2018; Newton, 1998). The farms on Hestmannøy provide a continuous supply of grain or food pellets and we speculate that the intensity of competition for resources may therefore be higher in the more unpredictable habitats on Træna, when population size is relatively larger (e.g., Dhondt, 2010; Pepke, Niskanen, et al., 2022). Food insecurity during early life may lead to shorter telomeres (Andrews et al., 2021). Similar negative effects of population density on TL have been observed in griffon vultures (*Gyps fulvus*, Gangoso et al., 2021) and Atlantic salmon (*Salmo salar*, McLennan et al., 2021), and in crowding experiments with mice (*Mus musculus*, Kotrschal et al., 2007).

We found some evidence for successful natal dispersers to have shorter telomeres prior to dispersal than non‐disperser, especially among males from the farm‐island (Hestmannøy, with only six dispersers out of 167 males, Figure 4). These analyses were limited by the relatively small number of dispersers. In the introduction, we suggested that short telomeres may inform a dispersal syndrome (pace‐of‐life), where bolder and faster‐lived individuals are more likely to disperse. Other explanations may underlie this observation, including TL being affected by environmental factors that also induce dispersal (e.g., habitat quality, Wilbourn et al., 2017), or influences individual quality. However, short telomeres have been correlated with bold, aggressive, pessimistic, or impulsive behavior in fish and birds (Adriaenssens et al., 2016; Bateson et al., 2015; Espigares et al., 2021). Increases in the level of glucocorticoids are linked to dispersal in birds (Belthoff & Dufty, 1998; Récapet et al., 2016; Silverin, 1997), but although Pegan et al. (2019) found a small negative effect of corticosterone treatment on TL in wild tree swallows (*Tachycineta bicolor*), this did not affect the age of initial departure from the natal site. Boonekamp et al. (2014) compared telomere loss within the first month of life among philopatric and dispersing jackdaws (*Coloeus monedula*), but did not find any differences; however, their study was limited by a small sample size (five dispersers out of 30 recruits). House sparrows are short‐distance dispersers (Tufto et al., 2005), and TL may not be a generally significant physiological indicator of dispersal capacity at the small scale of metapopulations. In contrast, metabolically demanding long‐distance migration or dispersal increases oxidative stress (Costantini et al., 2007) and may thus have direct negative impacts on TL, as observed in migratory birds (Angelier et al., 2013; Bauer et al., 2016; Schultner et al., 2014).

In several species, longer TL is associated with higher survival (Bichet et al., 2020; Crocco et al., 2021; Eastwood et al., 2019; Froy et al., 2021; Heidinger et al., 2021; Ilska‐Warner et al., 2019; van Lieshout et al., 2021; Wilbourn et al., 2018; Young et al., 2021, but see Vedder et al., 2017). We found no evidence for an association between TL and first‐year survival or mortality over the lifespan in house sparrows (Figures 3b and 4b). Perhaps early‐life TL is uncoupled from survival because of high extrinsic mortality of (primarily juvenile) house sparrows (Figure S3) not related to early‐life TL (e.g., Criscuolo et al., 2020; Eastwood et al., 2019; Wood & Young, 2019). Alternatively, house sparrows may be able to mitigate negative effects of short telomeres later in life through telomere maintenance (e.g., Vedder et al., 2017). Meillere et al. (2015) found a negative effect of stress induced by anthropogenic noise exposure on early‐life TL in house sparrows, but this did not affect fledgling survival. Pepke, Kvalnes, et al. (2022) found no associations between TL and first‐year survival in house sparrows from two populations that were part of a bidirectional artificial body size selection experiment. However, both short and long early‐life TL tended to be weakly associated with the lowest mortality rates over the lifespan in that study (Pepke, Kvalnes, et al., 2022), suggesting disruptive selection on TL. Furthermore, some studies have showed that early‐life TL was a poor predictor of survival, which was instead predicted by changes in TL (Boonekamp et al., 2014; Seeker et al., 2021; Wood & Young, 2019), which we did not measure in this study.

We found a tendency (i.e., with CIs still overlapping zero) for a negative association between ARS and TL even when accounting for the positive effect of body size on ARS, that is, individuals with short TL tended to produce more recruits annually (Figure 7b). This suggests that individuals with short early‐life telomeres may exhibit a faster pace‐of‐life reflected in higher ARS (and higher dispersal probability), while individuals with longer telomeres allocate more resources into self‐maintenance and hence a slower pace‐of‐life (Giraudeau, Angelier, & Sepp, 2019; Rollings, Friesen, et al., 2017; Young, 2018). Perhaps individuals with short TL therefore adopt a terminal investment strategy (Bauer et al., 2018; Clutton‐Brock, 1984), but as discussed above, we observed no association between TL and survival to suggest that TL is a causal mediator of trade‐offs between reproduction and survival (Young, 2018). Heidinger et al. (2021) found no associations between early‐life TL and annual reproductive performance (number of offspring) in wild American house sparrows. Instead, they found a positive relationship between early‐life TL and lifespan in females, but a negative trend between TL and lifespan in males. They therefore suggested that TL reflected differences in quality or condition in females, but did not predict pace‐of‐life (Heidinger et al., 2021). It will be interesting to see if other studies find contrasting associations between TL and fitness across different populations within the same species in the wild, and how TL is associated with other measures of pace‐of‐life not included in this study.

Early‐life TL has been shown to predict later‐life TL in some species (Froy et al., 2021; Martens et al., 2021), but there is also evidence that telomere loss rates are higher in longer telomeres (Atema et al., 2019; Atema et al., 2021; Verhulst et al., 2013; Victorelli & Passos, 2017). Alternatively, changes in TL in response to environmental variables through life (Brown et al., 2021; Chatelain et al., 2020) suggest that TL must be measured closer to reproduction events to better reveal associations with fitness (Marasco et al., 2021; Wood & Young, 2019). In this study, the large sample size and small amounts of available DNA made the high‐throughput qPCR the choice of method of TL measurements (Nussey et al., 2014). However, recent studies have indicated that qPCR TL data have lower and more variable within‐individual repeatability compared to the “golden standard” terminal restriction fragment (TRF) method (Bauch et al., 2021; Boonekamp et al., 2021; Kärkkäinen, Briga, et al., 2021; Vedder et al., 2021), which may be attributed to higher measurement error in qPCR (Morinha et al., 2020; Nettle et al., 2019). This may lead to less precision in associations between TL, environment, and fitness variables as evidenced across studies in recent meta‐analyses (Chatelain et al., 2020; Remot et al., 2021; Wilbourn et al., 2018). Future studies should seek to verify results of qPCR studies suggesting significant environmental effects on TL in wild populations to ensure that there is no publication bias towards strong associations.

Our study shows that environmental stressors negatively affected TL in young house sparrows. In contrast to our expectations, we found no fitness costs associated with shorter early‐life TL in the wild. Instead, we found some evidence that TL may be a biomarker of pace‐of‐life syndromes with fast‐paced individuals with short telomeres tending to have higher dispersal rates and higher ARS. Thus, there may be few long‐term physiological disadvantages associated with having short telomeres in early‐life in wild populations, but TL may rather act as a biomarker of individual pace‐of‐life. However, associations between early‐life TL, individual fitness, and complex environmental interactions seems difficult to establish and may vary between populations in the wild.

## AUTHOR CONTRIBUTIONS

**Michael Le Pepke:** Conceptualization (lead); data curation (lead); formal analysis (lead); investigation (lead); methodology (lead); project administration (supporting); resources (equal); validation (lead); visualization (lead); writing – original draft (lead); writing – review and editing (lead). **Thomas Kvalnes:** Conceptualization (supporting); data curation (equal); formal analysis (supporting); investigation (supporting); methodology (supporting); resources (equal); supervision (equal); writing – review and editing (supporting). **Peter Sjolte Ranke:** Data curation (equal); investigation (supporting); resources (equal); writing – review and editing (supporting). **Yimen Araya‐Ajoy:** Investigation (supporting); methodology (supporting); resources (equal); writing – review and editing (supporting). **Jonathan Wright:** Conceptualization (supporting); methodology (supporting); supervision (supporting); writing – review and editing (supporting). **Bernt‐Erik Sæther:** Conceptualization (supporting); funding acquisition (lead); project administration (equal); resources (supporting); writing – review and editing (supporting). **Henrik Jensen:** Conceptualization (supporting); funding acquisition (equal); methodology (supporting); project administration (equal); resources (equal); supervision (equal); writing – review and editing (supporting). **Thor‐Harald Ringsby:** Conceptualization (equal); funding acquisition (equal); project administration (equal); resources (equal); supervision (lead); writing – review and editing (supporting).

## CONFLICT OF INTEREST

The authors have no conflicts of interest.

### OPEN RESEARCH BADGES

This article has earned an Open Data badge for making publicly available the digitally‐shareable data necessary to reproduce the reported results. The data is available at https://doi.org/10.5061/dryad.612jm6463.

## Supporting information


Appendix S1

## Data Availability

Data is available on Dryad (https://doi.org/10.5061/dryad.612jm6463).
