# Supplementary material for: Causes and consequences of variation in early‐life telomere length in a bird metapopulation
Source: Ecol Evol. 2022 Jul 31;12(8):e9144. doi: 10.1002/ece3.9144 (PMC9339764; doi:10.1002/ece3.9144)
Supplement: Supplementary file 1 — Appendix S1 [file ECE3-12-e9144-s001.docx]

*Supporting information*

**Causes and consequences of variation in early-life telomere length in a bird metapopulation**

Michael Le Pepke^*^, Thomas Kvalnes, Peter Sjolte Ranke, Yimen G. Araya-Ajoy, Jonathan Wright, Bernt-Erik Sæther, Henrik Jensen & Thor Harald Ringsby

Centre for Biodiversity Dynamics (CBD), Department of Biology, Norwegian University of Science and Technology (NTNU), Trondheim, Norway

^*^Correspondence: Michael Le Pepke, email: michael@pepke.dk

**Contents**

[Sample sizes 2](#_Toc106565388)

[Telomere length variation across cohorts and population size fluctuations 3](#_Toc106565389)

[Sliding window analyses 3](#_Toc106565390)

[Dispersal probability and telomere length 6](#_Toc106565391)

[Fitness consequences of telomere length 7](#_Toc106565392)

[References 10](#_Toc106565393)

# **Sample sizes**

**Table S1:** Number of TL sampled fledglings in each cohort (year) used in this study, and island-wide population density (spring pre-breeding census of adults) for each island (Hestmannøy and Træna).

| **Cohort (year)** | **Hestmannøy**  **number of fledglings** | **Træna number of fledglings** | **Total number of fledglings)** | **Hestmannøy**  **population density (adults)** | **Træna**  **population density (adults)** |
| --- | --- | --- | --- | --- | --- |
| **1994** | 103 | - | 103 | 93 | - |
| **1995** | 90 | - | 90 | 86 | - |
| **1996** | 48 | - | 48 | 65 | - |
| **1997** | 81 | - | 81 | 65 | - |
| **1998** | 100 | - | 100 | 93 | - |
| **1999** | 90 | - | 90 | 130 | - |
| **2000** | 73 | - | 73 | 118 | - |
| **2001** | 41 | - | 41 | 114 | - |
| **2002** | 97 | - | 97 | 69 | - |
| **2003** | 106 | - | 106 | 94 | - |
| **2004** | 106 | 67 | 173 | 99 | 26 |
| **2005** | 95 | 90 | 185 | 136 | 86 |
| **2006** | 123 | 59 | 182 | 143 | 82 |
| **2007** | 152 | 127 | 279 | 188 | 74 |
| **2008** | 83 | 38 | 121 | 146 | 36 |
| **2009** | 182 | 35 | 217 | 160 | 44 |
| **2010** | 129 | 44 | 173 | 210 | 54 |
| **2011** | 248 | 65 | 313 | 240 | 50 |
| **2012** | 70 | 24 | 94 | 136 | 55 |
| **2013** | 93 | 87 | 180 | 97 | 59 |
| **2014** | - | - | - | - | - |
| **2015** | - | - | - | - | - |
| **2016** | - | - | - | - | - |
| **2017** | - | - | - | - | - |
| **Sum:** | 2110 | 636 | 2746 |  |  |

# **
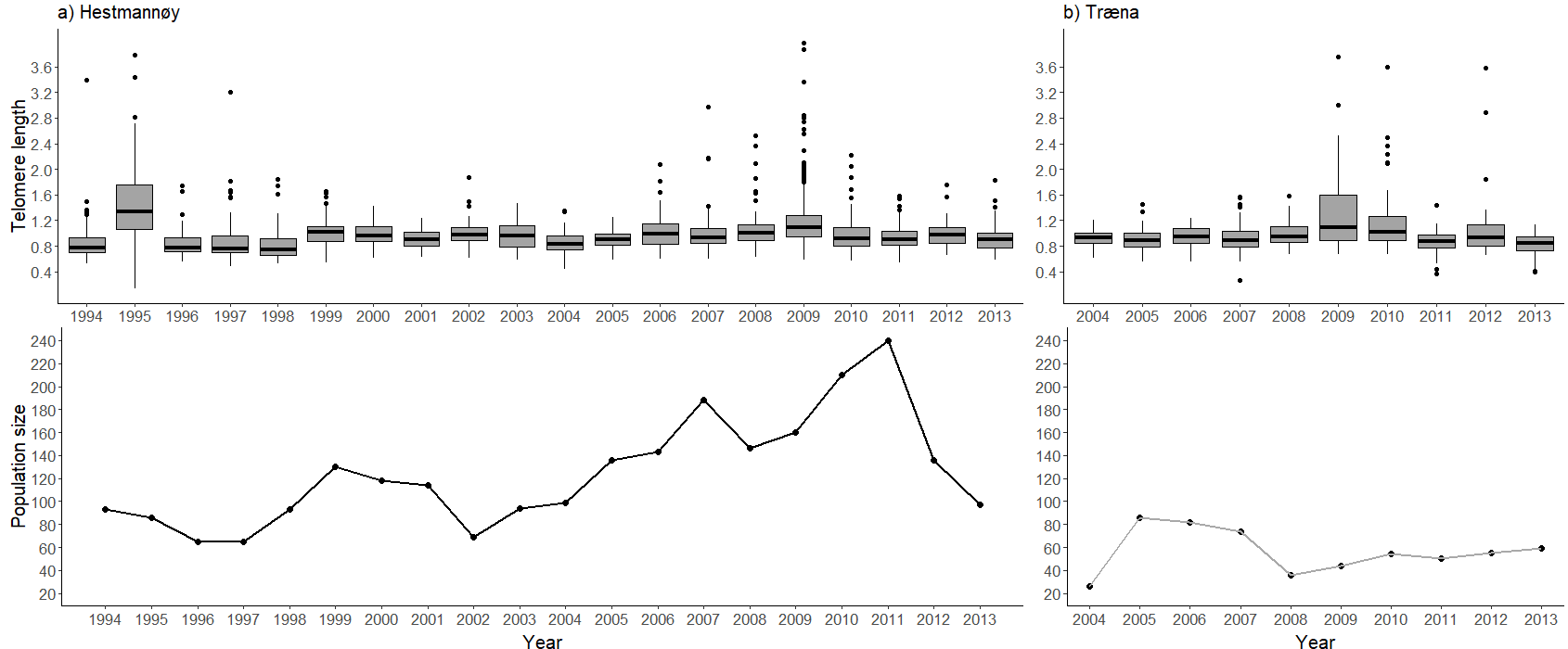
Telomere length variation across cohorts and population size fluctuations**

**Fig. S1:** Variation in individual early-life telomere length in house sparrow fledglings (*n*=2746) across all cohorts and the adult pre-breeding population size estimates for the two island populations a) Hestmannøy (1994-2013) and b) Træna (2004-2013).

# **Sliding window analyses**

**Table S2:** Results from sliding windows analyses of the effect of weather variables (temperature, precipitation, humidity, and the NAO index) on TL in house sparrow chicks. The daily NAO index is measured as the difference in atmospheric pressure at sea level between the Subtropical (Azores) High pressure and the Subpolar (Iceland) Low pressure. The sample size of *n*=2462 is higher than in Table 2 (*n*=2456) because individuals with missing body condition estimates could be included here. The best model (most sensitive timeframe) for each weather variable is shown with *ΔAICc* relative to a null (baseline) model containing no weather variables. The best performing combination is highlighted in bold, and all models with negative *ΔAICc* in italics. The baseline model was: log_10_(TL) ~ sex + tarsus + island*density, including year and brood identity as random intercepts. The third-best model (*ΔAICc*=-7.98) included a negative effect of maximum NAO 30-12 days prior to sampling and a significant interaction term between maximum NAO and island identity (*β_max. NAO 30-12 days_*=-0.040±0.009, CI=[-0.057, -0.023], *β_island (Hestmannøy)*max. NAO 30-12 days_*=0.024±0.010, CI=[0.005, 0.043]), providing some evidence that the negative effect of the weather variable was strongest on the no-farm island of Træna and significantly reduced on the farm island of Hestmannøy.

| **Model** | **Weather variable** | **Statistic** | **Function** | **Island interaction** | **ΔAICc** | **Window open** | **Window close** |
| --- | --- | --- | --- | --- | --- | --- | --- |
| 1 | temperature | mean | linear | no | 7.13 | 6 | 6 |
| 2 | pressure | mean | linear | no | 11.89 | 0 | 0 |
| *3* | *NAO* | *mean* | *linear* | *no* | *-3.61* | *30* | *6* |
| 4 | temperature | max | linear | no | 4.96 | 11 | 0 |
| 5 | pressure | max | linear | no | 11.89 | 0 | 0 |
| *6* | *NAO* | *max* | *linear* | *no* | *-12.82* | *30* | *16* |
| 7 | temperature | min | linear | no | 7.13 | 6 | 6 |
| 8 | pressure | min | linear | no | 11.24 | 13 | 3 |
| 9 | NAO | min | linear | no | 3.12 | 25 | 25 |
| 10 | temperature | sum | linear | no | 7.13 | 6 | 6 |
| 11 | pressure | sum | linear | no | 11.89 | 0 | 0 |
| 12 | NAO | sum | linear | no | 2.81 | 30 | 7 |
| 13 | temperature | mean | linear | yes | 18.75 | 11 | 11 |
| 14 | pressure | mean | linear | yes | 21.42 | 30 | 30 |
| 15 | NAO | mean | linear | yes | 2.32 | 30 | 7 |
| 16 | temperature | max | linear | yes | 17.09 | 13 | 0 |
| 17 | pressure | max | linear | yes | 18.69 | 30 | 24 |
| *18* | *NAO* | *max* | *linear* | *yes* | *-7.98* | *30* | *12* |
| 19 | temperature | min | linear | yes | 18.75 | 11 | 11 |
| 20 | pressure | min | linear | yes | 9.85 | 15 | 7 |
| 21 | NAO | min | linear | yes | 6.13 | 29 | 9 |
| 22 | temperature | sum | linear | yes | 18.75 | 11 | 11 |
| 23 | pressure | sum | linear | yes | 21.42 | 30 | 30 |
| 24 | NAO | sum | linear | yes | 8.02 | 30 | 30 |
| 25 | precipitation | mean | linear | yes | 9.13 | 24 | 17 |
| 26 | precipitation | max | linear | yes | 15.66 | 29 | 27 |
| 27 | precipitation | sum | linear | yes | 15.79 | 17 | 17 |
| 28 | precipitation | mean | linear | no | 5.94 | 29 | 8 |
| 29 | precipitation | max | linear | no | 7.29 | 17 | 17 |
| 30 | precipitation | sum | linear | no | 7.29 | 17 | 17 |
| 31 | temperature | mean | quadratic | yes | 45.32 | 15 | 12 |
| 32 | pressure | mean | quadratic | yes | 46.51 | 20 | 12 |
| 33 | NAO | mean | quadratic | yes | 13.93 | 30 | 9 |
| 34 | temperature | max | quadratic | yes | 45.97 | 15 | 14 |
| 35 | pressure | max | quadratic | yes | 46.61 | 16 | 3 |
| 36 | NAO | max | quadratic | yes | 2.88 | 26 | 12 |
| 37 | temperature | min | quadratic | yes | 42.18 | 29 | 12 |
| 38 | pressure | min | quadratic | yes | 42.97 | 19 | 12 |
| 39 | NAO | min | quadratic | yes | 17.4 | 29 | 1 |
| 40 | temperature | sum | quadratic | yes | 46.2 | 14 | 14 |
| 41 | pressure | sum | quadratic | yes | 54.58 | 13 | 13 |
| 42 | NAO | sum | quadratic | yes | 22.18 | 12 | 12 |
| 43 | temperature | mean | quadratic | no | 20.51 | 6 | 6 |
| 44 | pressure | mean | quadratic | no | 21.65 | 13 | 13 |
| *45* | *NAO* | *mean* | *quadratic* | *no* | *-0.41* | *25* | *9* |
| 46 | temperature | max | quadratic | no | 20.4 | 13 | 2 |
| 47 | pressure | max | quadratic | no | 18.12 | 16 | 3 |
| ***48*** | ***NAO*** | ***max*** | ***quadratic*** | ***no*** | ***-13.49*** | ***26*** | ***12*** |
| 49 | temperature | min | quadratic | no | 17.39 | 24 | 4 |
| 50 | pressure | min | quadratic | no | 21.65 | 13 | 13 |
| 51 | NAO | min | quadratic | no | 3.46 | 20 | 19 |
| 52 | temperature | sum | quadratic | no | 20.51 | 6 | 6 |
| 53 | pressure | sum | quadratic | no | 21.65 | 13 | 13 |
| 54 | NAO | sum | quadratic | no | 4.3 | 19 | 19 |
| 55 | precipitation | mean | quadratic | yes | 25.82 | 29 | 1 |
| 56 | precipitation | max | quadratic | yes | 46.49 | 28 | 27 |
| 57 | precipitation | sum | quadratic | yes | 47.15 | 18 | 18 |
| 58 | precipitation | mean | quadratic | no | 8.7 | 29 | 1 |
| 59 | precipitation | max | quadratic | no | 21.63 | 29 | 1 |
| 60 | precipitation | sum | quadratic | no | 26.8 | 21 | 21 |


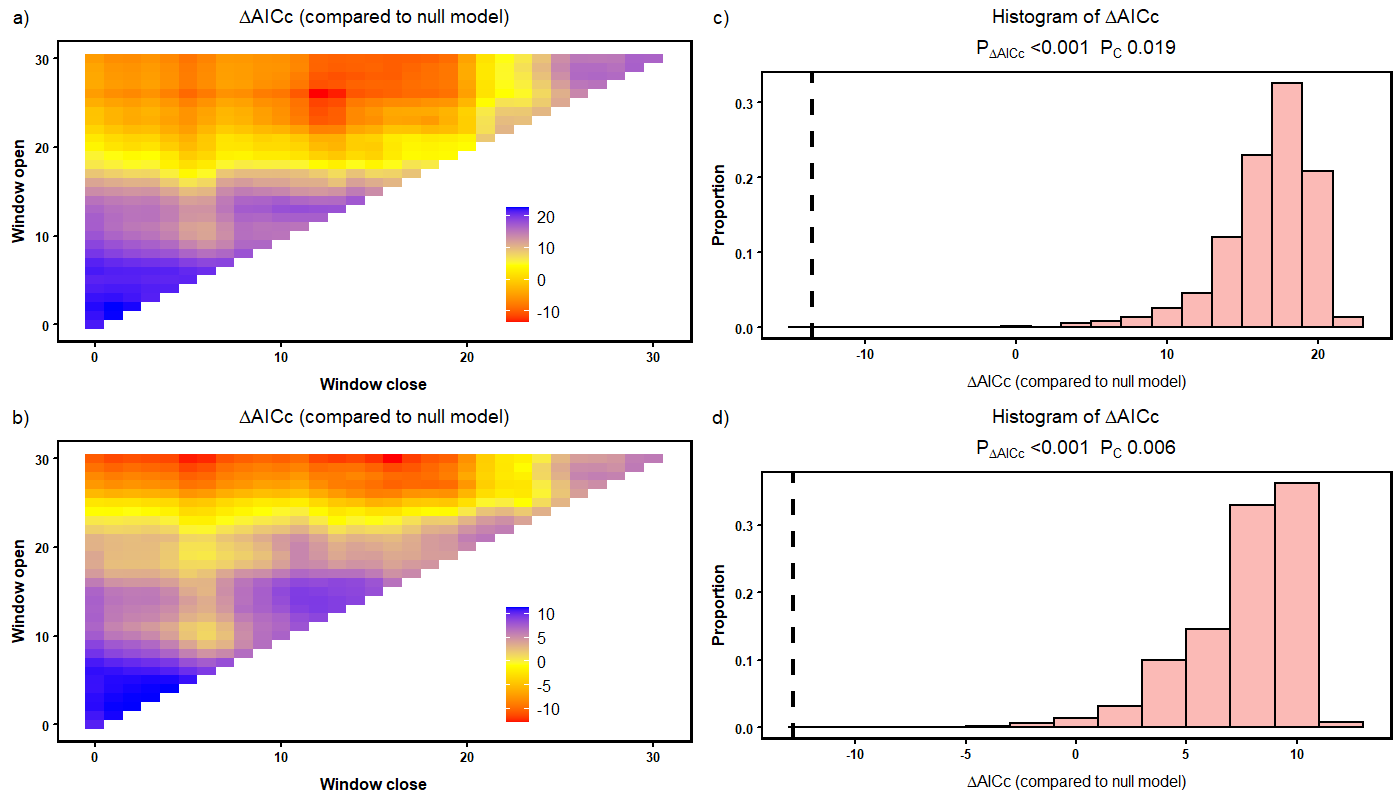
**Fig. S2: a and b)** Colours show the strength of the time window measured as ΔAICc (red are strong windows with low ΔAICc, blue are weak windows with high ΔAICc), i.e. how well the weather variable within a given timeframe improve a baseline model with no weather variables. Figure a shows the top model where the maximum NAO index is described with a linear function, and figure b shows the second-best model, where the maximum NAO index is described with a quadratic function. **c and d)** Tests for over-fitting using 500 randomisations of the data are compared to the real data (dotted line, where c: is a linear, and d: quadratic function for the maximum NAO index). Thus, neither model is likely to be statistical artefacts as a result of over-fitting (van de Pol et al., 2016).

**Table S3:** Pearson’s correlations coefficients between weather variables.

|  | **Precipitation** | **Pressure** | **NAO** |
| --- | --- | --- | --- |
| **Temperature** | *-0.0879*  *(p<0.0001)* | 0.0364  (p=0.0549) | 0.0340  (p=0.0733) |
| **Precipitation** |  | *-0.1447*  *(p<0.0001)* | *0.1314*  *(p<0.0001)* |
| **Pressure** |  |  | -0.0145  (p=0.4441) |

# **Dispersal probability and telomere length**

**Table S4:** AICc table of binomial generalized linear mixed models of variation in natal dispersal probability (*n*=445). All models included hatch year as random factor intercept. Models are ranked by AICc and shows number of degrees of freedom (df) and model weights (w).

|  | Model (*n*=445) | ∆AICc | df | w |
| --- | --- | --- | --- | --- |
| 1 | Dispersal = island + sex + island*sex | 0.0 | 5 | 0.195 |
| 2 | Dispersal = island + sex + island*sex + TL | 0.0 | 6 | 0.194 |
| 3 | Dispersal = island + sex + TL + island*sex*TL | 0.6 | 8 | 0.141 |
| 4 | Dispersal = island + sex | 0.9 | 4 | 0.127 |
| 5 | Dispersal = island + sex + TL | 1.2 | 5 | 0.108 |
| 6 | Dispersal = island + sex + TL + sex*TL | 1.5 | 6 | 0.091 |
| 7 | Dispersal = island + sex + TL + island*TL + sex*TL + island*sex | 2.1 | 8 | 0.068 |
| 8 | Dispersal = island + sex + TL + island*TL | 3.1 | 6 | 0.042 |
| 9 | Dispersal = island + sex + TL + island*TL + sex*TL | 3.5 | 7 | 0.034 |

**Table S5:** Estimates (*β*) with standard errors (SE) and lower and upper 95% confidence intervals (CI) from a binomial generalized linear mixed effects model of variation in natal dispersal probability (*n*=455). The model included a random intercept for hatch year. The three-way interaction effects are visualized in Fig. 3.

| Response variable: dispersal probability | *β* | SE | Lower CI | Upper CI |
| --- | --- | --- | --- | --- |
| intercept | 0.2927 | 1.0023 | -1.4094 | 2.6004 |
| sex (female) | -1.9067 | 1.4811 | -4.8867 | 0.9973 |
| island (Hestmannøy) | -1.2263 | 1.4939 | -4.2532 | 1.7031 |
| TL | 0.0538 | 0.7985 | -2.0403 | 1.3511 |
| TL * island (Træna) * sex (male) | -1.3294 | 1.2938 | -4.1242 | 1.3405 |
| TL * island (Hestmannøy) * sex (male) | -3.0495 | 1.7648 | -6.7867 | 0.1891 |
| TL * island (Træna) * sex (female) | -0.0338 | 1.5339 | -3.2599 | 2.9403 |
| σ^2^_year_ (*n*=20) | 0.4774 |  | 0.0509 | 1.7745 |
| Marginal R^2^ / Conditional R^2^: 0.256 / 0.350 | | | | |

# **Fitness consequences of telomere length**

|  | Model (*n*=2462) | ∆AICc | df | w |
| --- | --- | --- | --- | --- |
| 1 | Survival = sex + island + tarsus + tarsus^2^ | 0.0 | 7 | 0.280 |
| 2 | Survival = sex + island + tarsus | 0.3 | 6 | 0.240 |
| 3 | Survival = sex + island + tarsus + tarsus*island | 1.7 | 7 | 0.118 |
| 4 | Survival = sex + island + tarsus + tarsus^2^ + TL | 2.0 | 8 | 0.104 |
| 5 | Survival = sex + island + tarsus + TL | 2.3 | 7 | 0.089 |
| 6 | Survival = sex + island + tarsus + tarsus*island + TL | 3.7 | 8 | 0.044 |
| 7 | Survival = sex + island + tarsus + tarsus^2^ + TL + TL^2^ | 3.8 | 9 | 0.041 |
| 8 | Survival = sex + island + tarsus + TL + TL^2^ | 4.2 | 8 | 0.035 |
| 9 | Survival = sex + island + tarsus + TL + TL*island | 4.3 | 8 | 0.033 |

**Table S6:** Binomial generalized linear mixed models with ∆AICc<5 of variation in first-year survival probability (*n*=2462). Random factor intercepts for year (*n*=20) and brood identity (*n*=948) were included in all models. Models are ranked by AICc, and number of degrees of freedom (df) and model weights (w) are shown.

**Table S7:** AICc table of Cox hazard regression candidate models with ∆AICc<5. All models included sex as a fixed effect, brood identity as clusters, and was stratified by island identity. Models are ranked by AICc and shows number of degrees of freedom (df) and model weights (w).

|  | Model (*n*=2462) | ∆AICc | df | w |
| --- | --- | --- | --- | --- |
| 1 | Mortality = sex + strata(island) + tarsus + tarsus^2^ | 0.0 | 3 | 0.343 |
| 2 | Mortality = sex + strata(island) + tarsus | 1.1 | 2 | 0.195 |
| 3 | Mortality = sex + strata(island) + tarsus + tarsus^2^ + TL | 2.0 | 4 | 0.126 |
| 4 | Mortality = sex + strata(island) + tarsus + tarsus:strata(island) | 2.4 | 3 | 0.106 |
| 5 | Mortality = sex + strata(island) + tarsus + TL | 3.1 | 3 | 0.072 |
| 6 | Mortality = sex + strata(island) + tarsus + tarsus^2^ + TL + TL^2^ | 3.9 | 5 | 0.049 |
| 7 | Mortality = sex + strata(island) + tarsus + tarsus:strata(island) + TL | 4.4 | 4 | 0.039 |


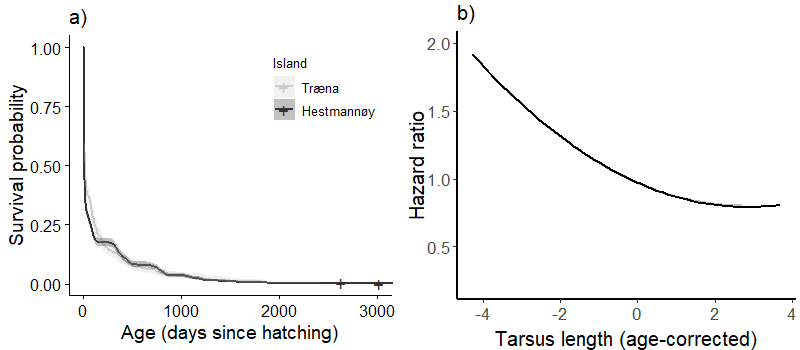


**Fig. S3:** Survival probability as a function of age in days since hatching shown separately for each island (Hestmannøy in black and Træna in grey). Crosses indicate timing of censoring for birds that may still be alive at the end of the data collection (*n*=2). There was no significant difference in mortality risk between the two strata (populations).

**Table S8:** AICc table of candidate models with ∆AICc<5 describing variation in annual reproductive success (ARS). All models included individual identity and year as random intercepts. Models are ranked by AICc and shows number of degrees of freedom (df) and model weights (w).

|  | Model (*n*=709) | ∆AICc | df | w |
| --- | --- | --- | --- | --- |
| 1 | ARS = sex + island + TL | 0.0 | 6 | 0.148 |
| 2 | ARS = sex + island + TL + tarsus | 0.1 | 7 | 0.144 |
| 3 | ARS = sex + island + tarsus | 0.6 | 6 | 0.111 |
| 4 | ARS = sex + island | 0.8 | 5 | 0.100 |
| 5 | ARS = sex + island + TL + tarsus + tarsus^2^ | 1.3 | 8 | 0.076 |
| 6 | ARS = sex + island + TL + TL*island | 1.9 | 7 | 0.058 |
| 7 | ARS = sex + island + tarsus + tarsus^2^ | 1.9 | 7 | 0.057 |
| 8 | ARS = sex + island + TL + TL^2^ | 1.9 | 7 | 0.056 |
| 9 | ARS = sex + island + TL + TL*island + tarsus | 1.9 | 8 | 0.056 |
| 10 | ARS = sex + island + TL + TL^2^ + tarsus | 2.0 | 8 | 0.053 |
| 11 | ARS = sex + island + TL + tarsus + tarsus*island | 2.1 | 8 | 0.052 |
| 12 | ARS = sex + island + tarsus + tarsus*island | 2.6 | 7 | 0.040 |
| 13 | ARS = sex + island + TL + TL^2^ + tarsus + tarsus^2^ | 3.3 | 9 | 0.028 |
| 14 | ARS = sex + island + TL + TL*island + tarsus + tarsus*island | 4.0 | 9 | 0.020 |

# **References**

van de Pol, M., Bailey, L. D., McLean, N., Rijsdijk, L., Lawson, C. R., & Brouwer, L. (2016). Identifying the best climatic predictors in ecology and evolution. *Methods in Ecology and Evolution, 7*(10), 1246-1257. doi:10.1111/2041-210X.12590
